# A Polarization-Multiplexed Multifunctional Metalens for Triple-Focus Coplanar Imaging

**DOI:** 10.3390/nano16040232

**Published:** 2026-02-11

**Authors:** Haiou Lu, Bing Yan, Jiyuan Zhang, Qian Zhou, Kai Ni, Min Zhang, Xiaohao Wang

**Affiliations:** 1 Department of Precision Instrument, Tsinghua University, Beijing 100084, China; lho16@tsinghua.org.cn; 2 Shenzhen International Graduate School, Tsinghua University, Shenzhen 518055, China; yan-b20@tsinghua.org.cn (B.Y.); zhangjiy25@mails.tsinghua.edu.cn (J.Z.); zhang.min@sz.tsinghua.edu.cn (M.Z.); wang.xiaohao@sz.tsinghua.edu.cn (X.W.); 3 State Key Laboratory of Precision Measurement Technology and Instruments, Department of Precision Instrument, Tsinghua University, Beijing 100084, China

**Keywords:** metalens, polarization-multiplexed, polarization imaging, multifunctional imaging

## Abstract

Metalenses capable of controlling more than two types of polarized light have drawn broad interest recently, as they can bring great flexibility and possibilities to the design of highly integrated polarization imaging devices that are not limited by theoretical efficiency. However, current metalenses for practical applications can only perform imaging with dual-polarization states simultaneously through a single aperture. Herein, a polarization-multiplexed multifunctional metalens enabled with three kinds of phase manipulation is proposed. Specifically, by encoding three groups of specific meta-atoms within a single aperture through spatial multiplexing, a “three-in-one” meta-device acting as a composite metalens can be constructed. Theoretical analysis and calculation simulations validate the performance of the metalens. Under circularly polarized light, the metalens exhibits three nearly diffraction-limited coplanar focal spots with horizontal, vertical, and circular polarization properties, respectively, and has a total focusing efficiency of 60%. This device can be used for triple-polarization imaging in real time, with promising prospects in highly integrated polarization imaging systems.

## 1. Introduction

Polarization is one of the important properties of light. Analyzing polarization properties of reflected, scattered, or transmitted light can reveal valuable information about the shape, texture, and even the constituent materials of a target object. For this reason, polarization imaging techniques that integrate polarization detection into imaging can effectively expand the dimensions of the acquired information [1,2,3,4]. Typically, polarization imaging systems can be divided into four groups: division of time, division of amplitude, division of aperture, and division of focal plane [5,6,7,8,9,10]. All of these techniques estimate the Stokes vector by measuring the intensity profile in different polarization bases. However, conventional division-of-time, -amplitude, and -aperture polarization imaging apparatus is usually bulky and requires many discrete optical elements [5,6,7], which limits its use for some complex environments. Division-of-focal-plane imaging polarimeters (DOFP-IPs) are more compact and contain fewer complicated optics compared to the three other categories. But the drawback of DOFP-IPs is that they suffer from a theoretical efficiency limit of 50% resulting from polarization filtering or spatial blocking [8,9,10].

Metasurfaces are a category of ultrathin artificially designed diffractive optical elements composed of subwavelength structural units, which can arbitrarily manipulate the amplitude, phase, and polarization of the incident light [11,12,13,14,15]. They are suitable for highly integrated and miniaturized optical systems due to their compatibility with conventional microfabrication techniques [16,17]. With judicious design, metasurfaces have been utilized for on-chip polarimetry [18,19] and polarization-sensitive imaging [20,21,22,23]. In contrast with the conventional polarization imaging equipment, a metasurface acting as a polarization-sensitive metalens is not only compact in size, but it can also simultaneously image the incoming scene with multiple polarization states through a single aperture, overcoming the 50% theoretical efficiency limit. However, current metalenses for practical applications can only image the incoming scene with orthogonal polarization states [24,25]. Moreover, such metalenses are generally realized by employing a polarization-multiplexed meta-atom or arranging different nanostructures to form an interlaced metasurface. The polarization-multiplexed meta-atom contains multiple variable geometric parameters. With limited degrees of freedom, simultaneously varying any parameters of a nanostructure would lead to unwanted optical responses. Therefore, the phase modulation of the incident light by the polarization-multiplexed units is not ideal. Aperture division and meta-atom replacement are general approaches to design interleaved metasurfaces [23,24], but these methods increase the types of polarization modulation by sacrificing the modulation effect, constraining the energy utilization. A strategy combining a polarization-multiplexed meta-atom and meta-atom replacement has also been proposed to achieve imaging of more polarization states [25], but it still presents the aforementioned problems. It is worth noting that significant progress has been made in metasurface-based polarization imaging in recent years, with major advancements achieved in areas such as full-Stokes imaging [26], wide-field-of-view imaging [27], sunlight polarimetric imaging [28], and noise-utilizing imaging [29].

In this work, a polarization-multiplexed multifunctional metalens is proposed that can be applied to simultaneously take images of three polarized scenes. The metalens consists of three groups of meta-atoms arranged in an aperture region by spatial multiplexing, realizing three independent phase manipulations. This metalens also behaves as an interlaced metasurface, but it is without aperture division and meta-atom replacement. Consequently, the space is fully leveraged to modulate more incident light, which improves energy utilization. Furthermore, all meta-atoms in the metalens are specific units containing only one parameter, and they can provide more precise phase shifts. The performance of the metalens is verified by numerical simulations. With the advantages of compactness, lightweight, and multi-polarization coplanar focusing, such a device can play an important role in highly integrated polarization imaging systems and may have promising applications in cloudy and foggy environments requiring linearly and circularly polarized imaging.

## 2. Metalens Design

**Figure 1:**
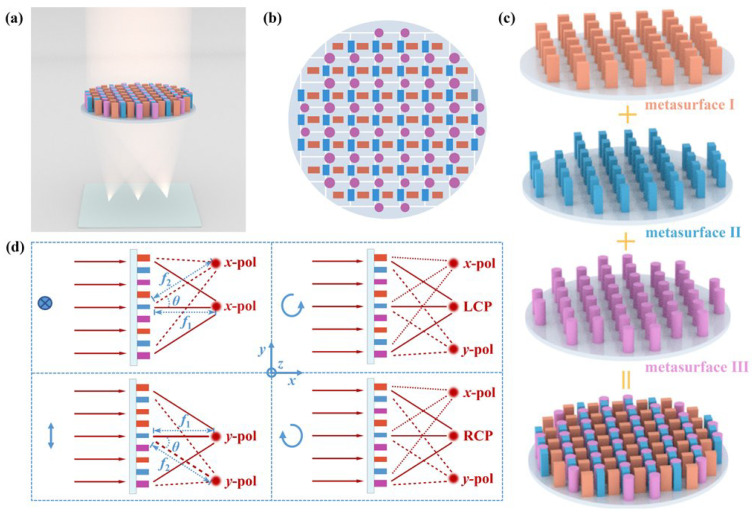
Design and polarization-modulated property illustration of the metalens. (**a**) Schematic diagram of the multifunctional metalens. (**b**) Top view of the designed metalens. The colors are used only to make the different meta-atoms easier to distinguish and do not carry any material information. (**c**) The construction scheme of the metalens. (**d**) Focusing diagrams of the metalens on differently polarized light. The arrows indicate the polarization direction of the incident light, representing horizontally polarized, vertically polarized, LCP, and RCP, respectively.

A metalens capable of producing three focal spots simultaneously in the same plane is presented, as shown in Figure 1a. This device consists of three families of silicon nanopillars located on a silica substrate. Figure 1b shows its top view, whereby different series of meta-atoms are alternately arranged on the substrate surface and the same ones are periodically connected by a square lattice. Each group of building blocks individually constitutes a metasurface, and then these metasurfaces are encoded by spatial multiplexing under one aperture to form a multifunctional composite metasurface, as shown in Figure 1c. In this way, when the coupling effect between the meta-atoms is weak, the composite metasurface can provide three independent phase profiles. Here, metasurface I, metasurface II, and metasurface III are designed for modulating horizontally, vertically, and arbitrarily polarized light, respectively. Such a metalens can be described by the Jones matrix as

(1) $$J\left(x,y\right)=\left(\begin{array}{cc} e^{i\varphi_{I}\left(x_{1},y_{1}\right)}+e^{i\varphi_{III}\left(x_{3},y_{3}\right)} & 0\\ 0 & e^{i\varphi_{II}\left(x_{2},y_{2}\right)}+e^{i\varphi_{III}\left(x_{3},y_{3}\right)} \end{array}\right)$$

where $\varphi_{I}\left(x_{1},y_{1}\right)$, $\varphi_{II}\left(x_{2},y_{2}\right)$ and $\varphi_{III}\left(x_{3},y_{3}\right)$ denote three uncorrelated phase profiles derived from metasurface I, metasurface II, and metasurface III in the metalens, and $\left(x_{1},y_{1}\right)$, $\left(x_{2},y_{2}\right)$ and $\left(x_{3},y_{3}\right)$ are the position coordinates of each metasurface’s building blocks.

**Figure 2:**
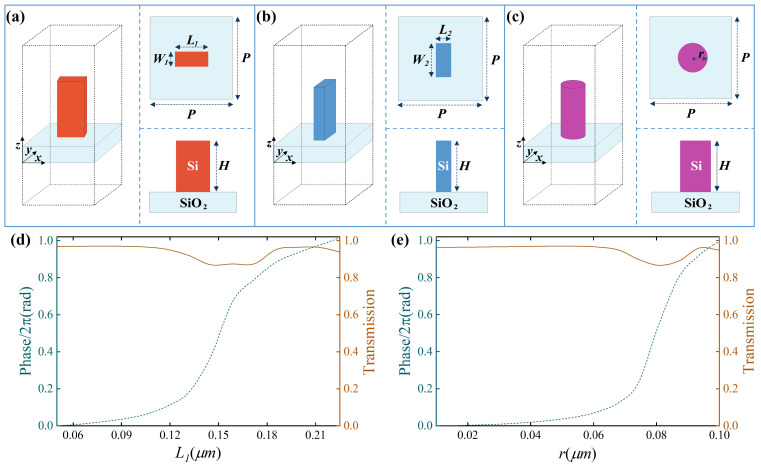
Meta-atom design of the metalens. (**a**–**c**) Perspective, top and side views of the three kinds of building blocks. The units in (**a**,**b**) have a fixed width of $W_{1}$ = 120 nm and length of $L_{2}$ = 120 nm, respectively. (**d**,**e**) Simulated phase shifts and transmission profiles with respect to the nanopillar length $L_{1}$ and radius *r*. Since the unit in (**b**) can be obtained by transposing the length and width of the unit in (**a**), its phase and transmittance with respect to width $W_{2}$ under y-polarized light are the same as the results of the unit in (**b**) with respect to length $L_{1}$ under x-polarized light.

For each polarization, light passing through the metalens is converged to different positions in the same plane. The required ideal phase curves of the three metasurfaces (I, II, and III) in the metalens take the forms

(2) $$\varphi_{I}\left(x_{1},y_{1}\right)=\frac{2\pi }{\lambda }\left(f_{1}-\sqrt{x_{1}^{2}+y_{1}^{2}+f_{1}^{2}+2x_{1}f_{1}\sin\theta }\right)$$

(3) $$\varphi_{II}\left(x_{2},y_{2}\right)=\frac{2\pi }{\lambda }\left(f_{1}-\sqrt{x_{2}^{2}+y_{2}^{2}+f_{1}^{2}-2x_{2}f_{1}\sin\theta }\right)$$

(4) $$\varphi_{III}\left(x_{3},y_{3}\right)=\frac{2\pi }{\lambda }\left(f_{2}-\sqrt{x_{3}^{2}+y_{3}^{2}+f_{2}^{2}}\right)$$

where λ is the design wavelength, θ is the off-axis angle, and $f_{1}$ and $f_{2}$ are the focal lengths of the metalens for differently polarized light ($f_{1}=f_{2}cos\theta$). Metasurface I and metasurface II are horizontal and vertical polarization-sensitive, respectively, and they follow a symmetric off-axis hyperbolic phase distribution. Metasurface III is polarization-insensitive with an on-axis hyperbolic phase distribution. Based on these design principles, in theory, when horizontally or vertically polarized light is incident, the metalens will produce two coplanar focal spots with the same polarization characteristics. Under the left-handed circularly polarized (LCP) or the right-handed circularly polarized (RCP) incident light, it will exhibit three coplanar focal spots with horizontal, vertical, and circular polarization properties, as shown in Figure 1d. Figure 2a–c depict the structural configurations of three distinct silicon nanopillar building blocks integrated on a fused silica substrate: (a) rectangular cross-section nanopillars with tunable length $L_{1}$ and fixed width $W_{1}$ equal to 120 nm, (b) rectangular cross-section nanopillars with variable length $W_{2}$ and fixed width $L_{2}$ equal to 120 nm, and (c) circular cross-section nanopillars with adjustable radius *r*. The fixed-width and variable-length rectangular nanopillars support specific waveguide modes with different effective refractive indices along the horizontal direction, which leads to horizontal polarization-dependent phase shifts. Similarly, the fixed-length and variable-width rectangular nanopillars can provide vertical polarization-dependent phase shifts. In contrast, the circular nanopillars are centrosymmetric, and they can generate specific waveguide modes effective in any direction as the radius varies. Thus they provide polarization-independent phase shifts. Since the nanostructures in the metalens need to be fabricated by single-step electron beam lithography, all the meta-atoms should be of the same height *H*.

In addition, to improve space utilization and ensure the integrity of each metasurface, their center-to-center spacing *P* should also be equal. Taking these factors into account, parametric sweeping of the structural units was conducted. When the center-to-center spacing *P* = 500 nm and the height *H* = 600 nm, the three groups of meta-atoms can satisfy the full phase coverage ranging from 0 to 2π while they all maintain a high transmittance close to 1. The corresponding transmittance amplitude and phase curves are shown in Figure 2d,e. The simulations were performed by using a finite-difference time-domain method (FDTD; the specific software employed was Ansys Lumerical 2020 R2.4) in the case of a normal incident plane wave with a free space wavelength of 800 nm. The refractive indices of Si and SiO_2_ are 3.69 and 1.45. Periodic boundary conditions were applied in the simulations. Mesh type was set to auto non-uniform, mesh accuracy was configured to 2, and mesh refinement was set to conformal variant 0.

## 3. Numerical Simulation and Verification

**Figure 3:**
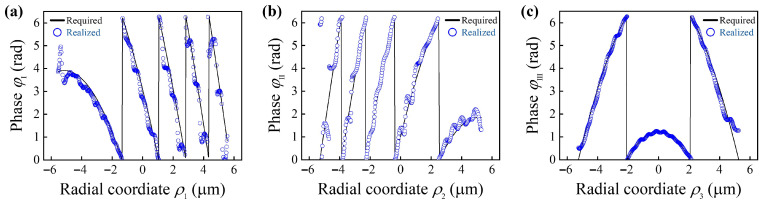
Phase matching diagram. (**a**–**c**) Realized (circular symbols) and required (solid curves) phase at each radial coordinate for metasurface I, metasurface II, and metasurface III of the metalens. Radial coordinate $\rho =\sqrt{x^{2}+y^{2}}$.

The metalens is designed to operate at the near-infrared wavelength of λ = 800 nm because its Rayleigh scattering loss is smaller than that of visible light in practical applications. The off-axis angle and focal lengths are set to θ = 20°, $f_{1}$ = 15 µm and $f_{2}$ = 14 µm. The radii of the three metasurfaces in the lens are 5.55 µm, 5.30 µm, and 5.25 µm and the three associated numerical apertures (NAs) are 0.35, 0.35, and 0.33, respectively. With the above parameter settings, the metasurface construction and phase distribution testing are conducted. The relationships between φ (i = I, II, III) and ($x_{j},y_{j}$), *j* = 1,2,3 are shown in Figure 3a–c. The solid curves and the circular symbols represent the required phases and the realized phases provided by the meta-atoms, respectively, which are in good agreement.

**Figure 4:**
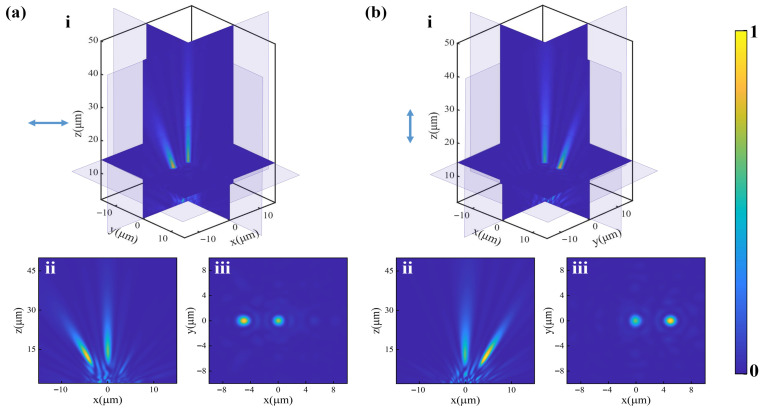
Simulated results for the metalens under (**a**) horizontally polarized light and (**b**) vertically polarized light. (**ai**,**bi**) Three-dimensional spatial slice maps of the intensity distribution on the transmission side. (**aii**,**bii**) Intensity distribution for the x–z planes through the center of the metalens. (**aiii**,**biii**) Intensity distribution at the respective focal planes of z = 14 µm.

The finite-difference time domain method was used to calculate the optical field modulation effect for differently polarized light. Figure 4(ai) shows the full-wave three-dimensional simulation results under horizontally polarized light. The intensity distribution in the x-z plane through the center of the metalens and in the desired focal plane of *z* = 14 µm are presented in Figure 4(aii,aiii). It can be seen that the horizontally polarized light passing through the metalens is focused at the expected positions, producing two focal spots. When the polarization of the incident light is switched to the vertical states, the incident light is also modulated into two convergent wavefronts, forming two focal points at different positions in the same plane, as shown in Figure 4b. As the polarization state of the incident light changes, the focal spot in the center of the focal plane always exists, while the focal spots on both sides will emerge or disappear depending on whether the incident light carries horizontal or vertical polarization components.

**Figure 5:**
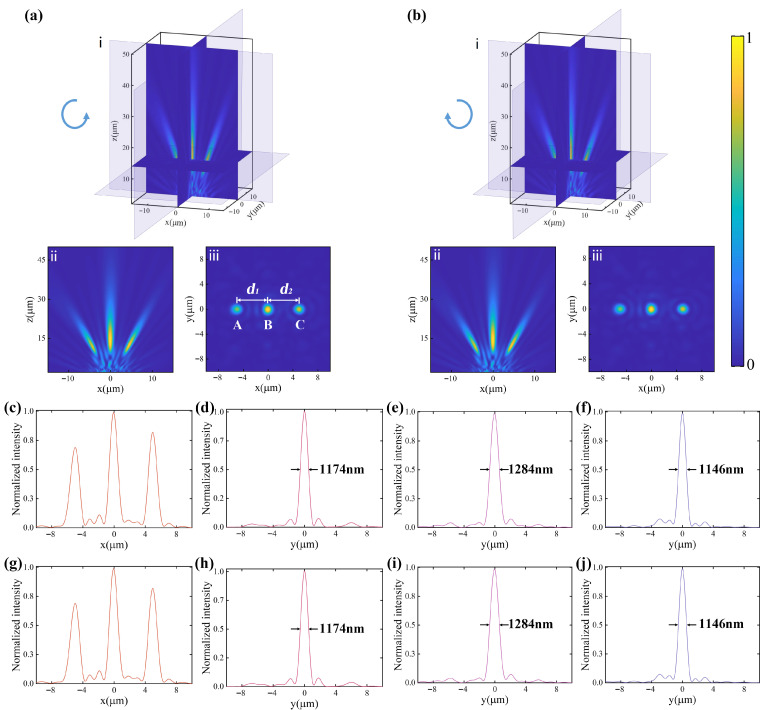
Simulated results for the metalens under (**a**) the LCP and (**b**) the RCP incident light. (**ai**,**bi**) Three-dimensional spatial slice maps of the intensity distribution on the transmission side. (**aii**,**bii**) Intensity distribution for the x–z planes through the center of the metalens. (**aiii**,**biii**) Intensity distribution at the focal plane of z = 14 µm. (**c**,**g**) Horizontal cuts of intensity profiles through the focal spots under the LCP and RCP light incidence, respectively. (**d**–**f**) and (**h**–**j**) Vertical cuts of intensity profiles for focal spots A, B, and C under LCP and RCP light incidence, respectively. The intensity distribution is normalized to the maximum intensity point in each curve and FWHMs of the focuses are labeled on the plots.

The modulation results of the metalens for LCP light are presented in Figure 5a. Among them, Figure 5(ai–aiii) show three-dimensional spatial slice maps of the intensity distribution on the transmission side, the intensity distribution in the x-z plane through the center of the metalens, and the intensity distribution in the desired focal plane of *z* = 14 µm, respectively.The metalens functions as a polarization recognition and focusing device here, converging the circular, horizontal and vertical polarization components of the incident light to the center and sides of the same plane. Likewise, the RCP light passing through the metalens is also modulated into three converging beams, as shown in Figure 5b. The distances between the three focal spots are d1 = 5.00 µm and d2 = 4.97 µm, and the associated actual off-axis angles are 19.65°and 19.54°, which are almost identical to the theoretical values. A better agreement can be implemented by further minimizing the phase matching error. Figure 5c shows a horizontal cut of the intensity distribution through the focal spots under the LCP light, and it can be seen that the intensities at the three focal spots are slightly different. In the case of constant focal lengths, the light intensity of each focal spot can be adjusted by changing the radius of the corresponding metasurface. However, varying the radius also affects the focusing efficiency and numerical aperture of the metalens. In the design, these factors have been taken into comprehensive account, resulting in minor variations in the relevant metrics of the three focal spots.

Figure 5d–f show the vertical cuts of intensity profiles for focal spots A, B, and C under LCP light; the full widths at half maximum (FWHMs) of the focal spots are about 1174 nm, 1284 nm, 1146 nm, respectively. The diameters of all the focal spots are on the scale of the operational wavelength, close to the diffraction limit. Symmetric beam profiles and nearly diffraction-limited focal size are related to the accuracy of phase realization. Focusing efficiency is defined as the ratio of the focal spot power to transmitted power through an aperture with the same radius as the metalens [30]. The simulated focusing efficiencies of the proposed device for horizontal, circular, and vertical polarizations of LCP light were calculated to be approximately 16%, 27%, and 17%, respectively, resulting in a total efficiency of 60%. The phase gradient on the metasurface is discontinuous and only light passing through the meta-atoms can be effectively modulated. The metalens designed here, acting as a “three-in-one” composite metasurface, makes full use of the space to enable more incident light to be manipulated. Therefore, compared with only a single metasurface under one aperture, it can increase the effective fraction of the transmitted light and improve the total energy utilization. Figure 5g–j show the horizontal and vertical cuts of intensity profiles through the focal spots under RCP light incidence, which are completely consistent with the results of the LCP incident light.

## 4. Conclusions and Prospect

In summary, a polarization-multiplexed multifunctional metalens was designed to simultaneously provide three phase gradients for modulating differently polarized light. The metalens was constructed based on a spatial multiplexing approach. This design scheme can both provide accurate phase modulation and improve energy utilization. The finite-difference time domain method was used to calculate the optical field modulation effect of the metalens. Horizontally or vertically polarized light passing through the metalens is modulated into two convergent wavefronts. For LCP or RCP light, the metalens exhibits three nearly diffraction-limited coplanar focal spots with a total focusing efficiency of 60%.

Looking forward, this lightweight and compact device is expected to image the input scene with three polarization states simultaneously to capture richer information, which is of interest for the development of metalens-based polarization imaging systems. Furthermore, circularly polarized light has a good polarization-maintaining effect and can enhance the system’s ability to penetrate clouds and fog. The performance, which can simultaneously realize linearly and circularly polarized light focusing, meets such a need; thus our metalens promises to advance the application of metalens-based polarization imaging systems in cloudy and foggy environments. The processing requirements of nanostructures have been fully considered in the design. Fabrication and imaging experiments of the metalens are in progress.

## Data Availability

The datasets generated and/or analyzed during the current study are available from the corresponding authors upon reasonable request.
